# A trait‐based plant economic framework can help increase the value of reforestation for conservation

**DOI:** 10.1002/ece3.8855

**Published:** 2022-04-29

**Authors:** Erik Petter Axelsson, Jane Vanessa Abin, Maria Lourdes T Lardizabal, Ulrik Ilstedt, Kevin C. Grady

**Affiliations:** ^1^ Wildlife Fish and Environmental Studies Swedish University of Agricultural Sciences Umeå Sweden; ^2^ Faculty of Tropical Forestry Universiti Malaysia Sabah Kota Kinabalu Malaysia; ^3^ Forest Ecology and Management Swedish University of Agricultural Sciences Umeå Sweden; ^4^ School of Forestry Northern Arizona University Flagstaff Arizona USA

**Keywords:** biodiversity, dipterocarpaceae, foundation species, plant traits, reforestation, tropical forest restoration

## Abstract

While reforestation is gaining momentum to moderate climate change via carbon sequestration, there is also an opportunity to use tree planting to confront declining global biodiversity. Where tree species vary in support of diversity, selecting appropriate species for planting could increase conservation effectiveness. We used a common garden experiment in Borneo using 24 native tree species to examine how variation among tree species in their support of beetle diversity is predicted by plant traits associated with “acquisitive” and “conservative” resource acquisition strategies. We evaluate three hypotheses: (1) beetle communities show fidelity to host identity as indicated by variation in abundance and diversity among tree species, (2) the leaf economic spectrum partially explains this variation as shown by beetle preferences for plant species that are predicted by plant traits, and (3) a small number of selected tree species can capture higher beetle species richness than a random tree species community. We found high variation among tree species in supporting three highly intercorrelated metrics of beetle communities: abundance, richness, and Shannon diversity. Variation in support of beetle communities was predicted by plant traits and varied by plant functional groups; within the dipterocarp family, high beetle diversity was predicted by conservative traits such as high wood density and slow growth, and in non‐dipterocarps by the acquisitive traits of high foliar K and rapid growth. Using species accumulation curves and extrapolation to twice the original sample size, we show that 48 tree species were not enough to reach asymptote levels of beetle richness. Nevertheless, species accumulation curves of the six tree species with the highest richness had steeper slopes and supported 33% higher richness than a random community of tree species. Reforestation projects concerned about conservation can benefit by identifying tree species with a disproportional capacity to support biodiversity based on plant traits.

## Graphical abstract

The functional relationship between plant diversity and diversity of organisms in higher trophic levels is a fundamental ecological question of what is important for understanding the contribution of restoration of plant communities for conservation. We show that tropical tree species vary in the level of diversity that they support and that this variation can be predicted by plant traits. We further illustrate how such variation can be utilized to increase the conservation value of plant diversity in restoration.

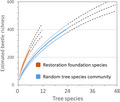

## 1 INTRODUCTION

Global reforestation efforts, such as those advocated by the Bonn Challenge and the Trillion Tree Campaign, are expanding at an increasing pace and at great cost to restore ecosystem services and to combat climate change through carbon sequestration. While the United Nations has proclaimed the current decade (2021–2030) as the decade of ecological restoration, there is still a fundamental lack of knowledge on how different tree species used in reforestation could be selected to facilitate a broader set of ecosystem functions such as promoting biodiversity. Incorporating the rebuilding of biodiversity as a cornerstone to the global reforestation movement could help battle global biodiversity loss (Barnosky et al., 2011; Ceballos et al., 2015) and the decline in diversity and abundance of insects referred to as the “insect apocalypse” (van Klink et al., 2020; Montgomery et al., 2020).

Selecting the appropriate tree species to include in reforestation is complicated in the global tropics as such ecosystems often contain thousands of tree species. It is logistically challenging to understand issues such as seed phenology and seedling dynamics that are important for designing plant propagation protocols of diverse species and, as such, reforestation efforts are often limited to only a few species (Chechina & Hamann, 2015). The selection of the few species that are generally involved in reforestation is typically based on what tree species are easily marketable as timber and what seeds are available (Brancalion et al., 2018). A recent meta‐analysis suggests that a large majority of tropical forest restoration projects in Southeast Asia use less than six species and a mean of three species (L. F. Banin, et al. in review). Furthermore, there is a growing global interest to use reforestation as a way to mitigate the consequences of climate change via carbon sequestration. In such approaches, there is a general preference to include fast‐growing tree species with acquisitive plant strategies resulting in fast growth and high potential for carbon sequestration. Hence, there is a clear need to assess in what way carbon sequestration of trees relates to other functions of reforestation (Locatelli et al., 2015; Paul et al., 2016).

Unsurprisingly, species variation in plant functional traits can be high in tropical forests (Brancalion et al., 2018; Charles, 2018; Gustafsson et al., 2016; Peters et al., 2016), and among different forest types these traits are useful for predicting functional and evolutionary strategies (Brancalion et al., 2018; Charles, 2018; Grady et al., 2013; Kursar et al., 2009). Two orthogonal axes of leaf and stem economic traits, both describing a continuum of “acquisitive” to “conservative” resource acquisition strategies, are useful for understanding plant function (Baraloto et al., 2010). The leaf economics spectrum reflects a trade‐off between carbon investment in productive leaves with rapid turnover vs. costly physical leaf structures with a long revenue stream. A second axis of variation, the “stem economics spectrum,” defines a similar trade‐off at the stem level: dense wood reflecting conservative resource use and slow growth vs. soft wood reflecting acquisitive strategy and fast growth (Chave et al., 2009). Importantly, these two axes of leaf and stem economic continuum are sometimes orthogonal (Baraloto et al., 2010), suggesting that traits and hence plant function can vary independently at the leaf and at the stem levels. There is also a growing literature demonstrating the role that functional variation among tree species may play in achieving specific reforestation goals; such as promoting plant establishment and growth, carbon sequestration, facilitating natural regeneration, increasing drought tolerance, or increasing plant diversity (Axelsson et al., 2020; Charles, 2018; Meli et al., 2013). However, there is still a fundamental lack of knowledge on how such variation may help to promote overall ecosystem biodiversity during tropical forest restoration (but see; Plath et al., 2012). Despite the overarching hypothesis that high plant diversity supports high diversity of associated arthropods and other taxonomic groups reliant on plant resources, there is little research concerned with *how* different plant species support different levels of arthropods diversity in a predictive context.

Given that there are logistical constraints that limit the number of tree species used in reforestation projects, there is a clear value to identify tree species with a disproportionate positive influence on biodiversity and to assess if the acquisitive–conservative trait continuum can help predict patterns of associated biodiversity. Organisms inhabiting the foliage of tree canopies may be particularly sensitive to variation in foliar traits associated with the leaf economic spectrum as these same traits limit resource use. Generally, it is thought that acquisitive traits are associated with high herbivory rates not only due to high nutrient levels that optimize resource quality for herbivores but also due to covariance between high nutrient levels and fast growth rates, where many arthropod taxa respond to plant vigor (Abdala‐Roberts et al., 2018; Price, 1991; Pringle et al., 2011). In addition, tropical tree species often vary in susceptibility to herbivory (Axelsson et al., 2020) suggesting that some species may attract more herbivores. There is evidence suggesting that variation in plant traits can predict the composition of herbivore communities with some groups of herbivores preferring highly nutritious leaves with high nutrient levels (Ohmart et al., 1985) and others simply preferring plants with fast growth rates (Abdala‐Roberts et al., 2018; Price, 1991; Pringle et al., 2011). Furthermore, the foundation species concept suggests that different tree species may vary disproportionally in value for supporting associated biodiversity (Ellison et al., 2005). Hence, if some tree species in tropical forests are found to have a disproportional positive effects on associated biodiversity, such species could potentially help in rebuilding biodiversity during reforestation. A relationship between biodiversity and acquisitive–conservative traits would be particularly valuable for restoration projects since these traits are commonly available from literature and easy to measure.

Although forest canopies are known to harbor a significant part of insect biodiversity found in hyper‐diverse tropical forests, among‐species variation in their ability to promote these communities is not well known (but see; Basset, 1999; Novotny et al., 2010; Plath et al., 2012; Stork, 1991). Findings of low host specificity of many insect taxa in tropical forests suggest that certain insect groups can sustain themselves on different hosts (Basset, 1999; Novotny et al., 2002). Novotny et al. (2002) found that insect herbivore communities across different hosts typically shared a third of their species suggesting that different plant species to some extent may fill similar functions, that is, can support similar communities. Furthermore, in tropical forests of Uganda, Wagner (2000) found that communities of canopy‐dwelling species differed little among different tree species. Nevertheless, there might also be patterns of host preference where certain taxa of herbivores may all cue in on the same traits, and if so, plants expressing those traits are more likely to attract a range of herbivores and thus support higher richness. Tropical tree species do vary in susceptibility to herbivory (Axelsson et al., 2020; Cárdenas et al., 2014) and it is possible that this preference may be dictated by traits (Cárdenas et al., 2014). In one of the first attempts to assess how tree species used in reforestation vary in canopy communities, Plath et al. (2012) found that three native tree species used in afforestation in Panama differed significantly in the composition of beetles they supported. Nevertheless, there is also evidence showing that functionally different groups of insets may cue in on different plant characteristics, that is, specialists herbivores may be more responsive to defensive chemistry, whereas generalists may cue in to the overall nutritional status of the plant (Volf et al., 2015).

Given these uncertainties, restoration managers today are not able to make informed decisions on how many tree species are needed to support biodiversity in tropical forests, or if there are tree species with certain traits that could be of particular value. Clearly, “acquisitive” plant strategies may be beneficial in reforestation focusing on carbon sequestration as it may promote resource acquisition and plant growth. However, despite that the plant economic spectrum is put forward as a universal driver of species co‐existence (Harrison & LaForgia, 2019; Pérez‐Ramos et al., 2019), ecosystem processes (de la Riva et al., 2019), and interactions with soil biota (Cowan et al., 2021; Shi et al., 2020), there have been no previous attempts to explicitly test in what way “acquisitive” plant strategies preferred in reforestation may link to biodiversity in higher trophic levels. There is, however, evidence showing that some of the plant traits linked to leaf economic spectrum may indeed be important for understanding how herbivores and herbivore communities interact with plants (Abdala‐Roberts et al., 2018; Ohmart et al., 1985; Price, 1991; Pringle et al., 2011). Understanding how this may translate to influence broader patterns of associated biodiversity could dictate a tree species contribution for supporting biodiversity during reforestation. This lack of information is serious since restoration programs today often focus on only a few fast‐growing species in favor of rapid establishment but lack understanding of the potential of these species to promote biodiversity. For example, if tree species with acquisitive plant strategies with fast growth also are preferred by diversity, this would mean that managers could emphasize primarily fast‐growing trees both for carbon sequestration and conservation, but if slow‐growing trees are more important it would make an important incentive to also include such species in a species portfolio.

The objectives of this study were to assess to what extent native tree species typically used in enrichment plantings in Borneo vary in their ability to promote biodiversity, and to assess in what way functional plant strategies along the acquisitive–conservative trait continuum influence the level of associated biodiversity that tree species support. We also aimed to assess the functional relationship between plant species richness and higher taxonomic level biodiversity and hence estimate the level of plant diversity needed to rebuild canopy insect diversity in degraded secondary tropical forests. We conducted our study within an 18,500 hectare experimental forest in Sabah, Malaysia – the INIKEA Sow‐a‐seed project – where enrichment plantings with more than 3 million trees from 80 species have been conducted, in part, to restore local plant biodiversity. Within this forest, we established a common garden where plants were propagated in a relatively homogenous environment within a secondary forest that allowed us to examine among‐species variation within a standard environment. Within the garden, a randomly selected group of 24 tree species was used to sample beetle communities that were collected at four separate sampling periods over 1 year in 2017–2018 when trees were 8 years old. The taxonomically and functionally diverse beetles (Coleoptera) were specifically chosen in this study due to their high diversity in the canopy of tropical forests (Chung et al., 2001). Beetles account for roughly 25% (350,000–400,000 species) of all described species (~1.5 million species) and 40% of all described insect species, making this the most species‐rich taxonomic group on Earth (Hammond, 1992; Stork et al., 2015) and include species of various functional roles such as predators, fungivores, detritivores, and herbivores. We specifically addressed three hypotheses: (1) beetle communities show fidelity to host identity as indicated by variation in abundance and diversity among tree species, (2) the leaf economic spectrum partially explains this variation as shown by beetle preferences for plant species that are predicted by plant traits, and (3) a small number of carefully selected tree species can capture higher beetle species richness than a random tree species community. Together this would demonstrate the conservation value of identifying tree species with a disproportionate capacity for supporting biodiversity as a framework for rebuilding biodiversity during restoration or reforestation of degraded tropical forests.

## 2 METHODS

### 2.1 Research site

**FIGURE 1:**
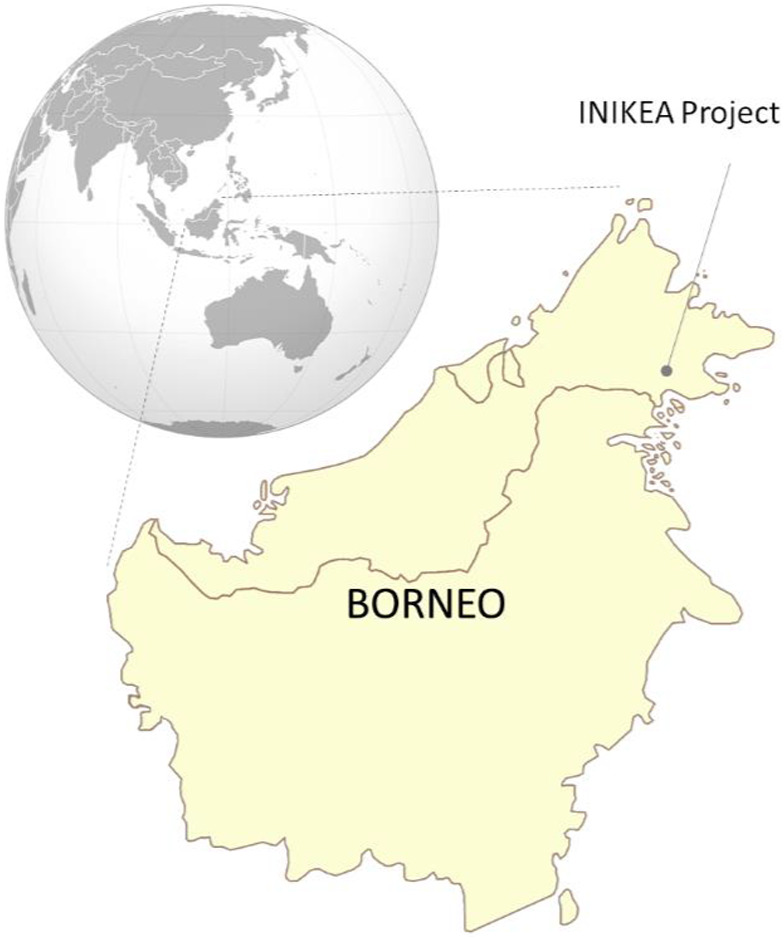
Location of the INIKEA restoration project in the northern Sabah State of Malaysian Borneo

Our research was conducted in the INIKEA Sow‐a‐seed restoration project, an 18,500 ha restoration project, including reforestation with over 3 million planted trees since the start of the project in 1998. The project area includes lowland dipterocarp forests in the Malaysian state of Sabah on the Island of Borneo (N 4.6 N, 117.2, elevation ~300 masl; Figure 1). Previously, the forest in the INIKEA site was subjected to intense logging between 1970 and 1980 and further degraded by wildfire in 1982 and 1983. The remaining secondary forest held different levels of disturbance, from heavily disturbed areas with a large element of pioneer species and weeds, to small patches of more or less pristine forest. The main aim of the project was to restore biodiversity through enrichment planting with a diverse selection of tree species; more than 80 native tree species have been used since project initiation (Gustafsson et al., 2016). The climate in the area is humid tropical equatorial with high precipitation throughout the year and moderately more precipitation between October and February (Peel et al., 2007). Mean annual precipitation measured at a weather station in Luasong approximately 5 km from the common garden was 2565 mm (SD 338 mm) for the years 2004 to 2016 (Axelsson et al., 2020).

### 2.2 Common garden

In 2008, we established a 3‐ha experimental common garden with 34 tree species of dipterocarps (an inordinately biodiverse plant family in Borneo that composes the majority of mature plant canopies), non‐dipterocarps, and fruits tree. The included species were selected to represent species that are commonly used in reforestation efforts in the mixed dipterocarp forests of Borneo, the INIKEA project area, and throughout the state of Sabah. The experimental common garden was established in the interior of the INIKEA project area, at an elevation of approximately 300 m. In the garden, 20 replicated trees of each of the 34 species, for a total of 680 seedlings, were randomly planted along lines separated by 10 m, and along lines, trees were planted at 3 m intervals. The plant material used to establish the garden came from seeds collected within the INIKEA project forest, germinated in the project nursery, and were of approximately equal age and height when out‐planted. All competing vegetation was removed in the lines prior to planting, for example, ground vegetation, bushes, small pioneer trees, and climbers. We performed weed maintenance to keep the lines free from competing vegetation when necessary, resulting in 1–3 rounds of weeding per year corresponding to the INIKEA standard procedure for restoration planting. More details on the experimental design can be found in Gustafsson et al. (2016).

### 2.3 Biodiversity assessment on different tree species

We randomly selected 24 species, of the 34 tree species available, comprising 17 dipterocarp and 7 non‐dipterocarp trees, to be included in this study (Table 1). Five replicate trees were randomly chosen from each of the 24 tree species. Four of the selected trees died over the course of our study, resulting in *Sindora irpicina* and *Shorea parvifolia* represented by four replicate trees, and *Walsura pinnata* was represented by three replicate trees. We sampled beetles using canopy fogging with a pyrethrum‐based insecticide. To account for seasonal variation in beetle occurrence and assess the overall contribution to biodiversity, we repeated sampling every 3 months for a total of four sampling periods between May 2016 and April 2017. For each selected tree and sampling period, a white canvas sheet (2 × 2 m) was laid on the ground below the tree canopy. We sprayed the insecticide for approximately 30 seconds with the fogging zone including the entire canopy of each tree (all 8‐year‐old trees were less than 10 m in height and easily reached with orchard ladders). For taller trees, we used an orchard ladder to reach the upper part of the canopy. After fogging, we waited 30 s for the insecticide to take effect and then vigorously shook the tree from the tree bole for 15 s to help detach arthropods from the canopy. Arthropods falling on the canvas were collected using forceps for 15 person minutes following fogging and immediately placed in vials containing an aqueous solution of 70% ethanol. The same procedure was repeated on all trees across sampling dates. We identified beetles to the species level where possible; all species were sorted to at least the family level which allowed us to categorize beetles into functional groups for some of the families. Richness estimates were based on both species identification and by morpho‐species classification. The use of morpho‐species generally provides a conservative estimate of richness as certain groups of beetles may sometimes be morpho‐typed as the same species. However, morpho‐typing is necessary for ecological studies as identifying specimens to species level where there are thousands of species is logistically difficult and beyond the scope of our research funding. Also, there are few taxonomic experts in the region and many insects in Borneo have not yet been described, which is a common problem throughout the hyper‐diverse tropics (Derraik et al., 2002). Specimens were compared with voucher specimens housed in collections at the Institute of Tropical Biology and Conservation (ITBC) located at University Malaysia Sabah (UMS) and the Forest Research Centre (FRC) in Sandakan, Sabah. Other references used for beetle identification include keys by Lawrence and Newton (1995), Lawrence et al. (2000), and Chung (2003). Specimen collections are stored at the Entomology lab, University Malaysia Sabah.

**TABLE 1:** Abundance and richness (in brackets) of beetles recorded on 24 tree species according to six feeding guilds

Function/Family	Tree species																								
	Shorea fallax	Shorea macroptera	Dipterocarpus conformis	Parashorea tomentella	Pentace adenophora	Shorea leprosula	Shorea ovalis	Mangifera odorata	Diospyros sp.	Hopea ferruginea	Dryobalanops lanceolata	Sindora irpicina	Shorea macrophylla	Shorea beccariana	Eugenia sp.	Parashorea symthiesii	Parashorea malaanonan	Shorea parvifolia	Pentace laxiflora	Shorea falciferoides	Shorea leptoderma	Dryobalanops keithi	Walsura pinnata	Canarium sp.	Total
Herbivores																									
Apionidae															2 (2)							1 (1)		1 (1)	4 (2)
Attelabidae	1 (1)						1 (1)			2 (2)	1 (1)			1 (1)			1 (1)		1 (1)				1 (1)		9 (4)
Chrysomalidae	25 (16)	15 (9)	24 (19)	12 (12)	17 (17)	6 (6)	54 (31)	13 (9)	38 (24)	40 (27)	18 (12)	15 (9)	9 (8)	16 (13)	23 (13)	24 (21)	32 (20)	4 (4)	36 (9)	25 (17)	46 (19)	14 (12)	14 (12)	20 (17)	540 (127)
Curculionidae	14 (10)	8 (6)	15 (9)	10 (7)	8 (8)	6 (4)	11 (9)	14 (6)	19 (10)	28 (12)	12 (8)	6 (3)	5 (3)	8 (6)	12 (7)	20 (15)	8 (6)	4 (4)	16 (10)	10 (6)	19 (11)	18 (8)	4 (3)	7 (7)	282 (57)
Elateridae	8 (6)	4 (3)	4 (4)	2 (2)		2 (2)	5 (3)	1 (1)	9 (3)	6 (5)	1 (1)		3 (2)	2 (2)	3 (2)	12 (2)	5 (3)		6 (5)	5 (3)	6 (3)	5 (4)	6 (6)	3 (2)	98 (29)
Lycidae	6 (3)	11 (5)	4 (3)	2 (2)	1 (1)		9 (9)	1 (1)	3 (3)	8 (5)		1 (1)	7 (5)	4 (4)	3 (3)		2 (2)		17 (5)	5 (4)	10 (5)	3 (2)	2 (2)	4 (3)	103 (25)
Mordellidae	2 (2)	2 (2)			1 (1)		1 (1)	2 (1)	2 (1)	1 (1)	1 (1)	1 (1)		1 (1)							2 (2)	2 (1)	3 (2)		21 (8)
Psephenidae		1 (1)	3 (1)																						4 (1)
Predators																									
Cantharidae			2 (1)				1 (1)		1 (1)	2 (1)	2 (2)	1 (1)		1 (1)			1 (1)		2 (1)			1 (1)			14 (2)
Carabidae	2 (2)	1 (1)		2 (1)		4 (4)	5 (4)	1 (1)	2 (2)		1 (1)			1 (1)	2 (2)	1 (1)	1 (1)	1 (1)	6 (2)		3 (2)	1 (1)			34 (14)
Cicindelidae		1 (1)	1 (1)	1 (1)						1 (1)			1 (1)		1 (1)										6 (3)
Coccinelidae		2 (1)	5 (5)	1 (1)			2 (2)	1 (1)	1 (1)	2 (1)	3 (2)				1 (1)		1 (1)	2 (1)	1 (1)	2 (1)		1 (1)	2 (1)		27 (15)
Lampyridae									1 (1)		1 (1)														2 (2)
Pselaphidae		1 (1)				1 (1)			2 (2)		1 (1)								1 (1)	1 (1)					7 (4)
Fungivores																									
Anthribidae		1 (1)							1 (1)		1 (1)	1 (1)													4 (2)
Cerylonidae	1 (1)																								1 (1)
Corylophidae	1 (1)											1 (1)		1 (1)											3 (1)
Endomychidae	1 (1)	1 (1)				1 (1)			1 (1)		1 (1)					1 (1)			1 (1)	1 (1)					8 (4)
Erotylidae																					1 (1)	1 (1)			2 (2)
Leiodidae			1 (1)		1 (1)				1 (1)																3 (2)
Ptilodactylidae					1 (1)				2 (1)		1 (1)									1 (1)				1 (1)	6 (2)
Scaphiididae						1 (1)													3 (1)						4 (1)
Saprophagous																									
Aderidae							1 (1)		1 (1)								3 (1)								5 (2)
Anthicidae			2 (2)	1 (1)			1 (1)		2 (2)	3 (3)			1 (1)				5 (4)		6 (3)		4 (4)		1 (1)	1 (1)	27 (17)
Geotrupidae				1 (1)																					1 (1)
Hybrosidae	1 (1)	1 (1)	1 (1)	1 (1)	1 (1)	1 (1)	3 (2)	1 (1)	5 (1)	2 (1)	3 (1)	4 (2)	2 (2)	2 (1)	1 (1)	1 (1)	3 (2)	1 (1)		3 (1)	1 (1)	3 (1)	1 (1)	1 (1)	43 (3)
Pedilidae		1 (1)					1 (1)													1 (1)					3 (1)
Ptinidae			2 (1)	1 (1)						2 (1)						1 (1)			1 (1)	1 (1)	3 (2)				11 (3)
Scirtidae													1 (1)			1 (1)					1 (1)				3 (1)
Xylophagous																									
Anobiidae			1 (1)				1 (1)		1 (1)	4 (4)		1 (1)	1 (1)	1 (1)			2 (2)		3 (1)						15 (6)
Brentidae								2 (2)				1 (1)			1 (1)		1 (1)				2 (1)		1 (1)		8 (5)
Cerambycidae	1 (1)	1 (1)	1 (1)	1 (1)		5 (5)			2 (2)	2 (2)	1 (1)	1 (1)		1 (1)	1 (1)			2 (1)	2 (2)	2 (2)	3 (2)	2 (1)			28 (19)
Lucanidae																1 (1)	1 (1)							1 (1)	3 (2)
Passalidae							1 (1)																		1 (1)
Polyphagous																									
Eucnemidae	3 (1)	2 (2)	2 (2)	1 (1)			1 (1)		1 (1)	6 (2)							1 (1)		1 (1)		2 (1)		2 (2)	1 (1)	23 (7)
Lagriidae											2 (1)								1 (1)						3 (2)
Meliolidae																	1 (1)								1 (1)
Nitidulidae				1 (1)						1 (1)															2 (2)
Nosodendridae																			2 (1)						2 (1)
Phalacridae			2 (1)						3 (2)												1 (1)		1 (1)		7 (2)
Scarabidae	1 (1)		1 (1)				1 (1)			2 (1)							1 (1)					1 (1)		1 (1)	8 (6)
Tenebrionidae	2 (2)	3 (3)	3 (3)	3 (3)	3 (2)	1 (1)	1 (1)		2 (2)	5 (4)					1 (1)	1 (1)	2 (2)		7 (4)	3 (3)		1 (1)	1 (1)	2 (2)	41 (15)

Note

The classification of families is based on Lawrence and Newton (1995), Lawrence et al. (2000), and Chung (2003).

### 2.4 Plant traits

To functionally describe our collection of tree species based on the plant economic spectrum, we compiled five species traits that are well‐known to dictate plant species position along the acquisitive–conservative trait continuum: foliar phosphorous (P), nitrogen (N), and potassium (K) concentrations (mass‐based), specific leaf area (SLA), and wood density (WD). Plants with high nutrient concentrations, high SLA, and low‐density wood tend to be correlated with fast resource acquisition and rapid growth (Baraloto et al., 2010). Species mean trait values for N, P, and K and SLA were compiled from an earlier study conducted in the same common garden (Gustafsson et al., 2016) on the same trees used in our study. Wood density was also derived from Gustafsson et al. (2016) and based on a local review of wood density values published in research literature. In addition to these functional traits estimates, we also assessed variation in realized height growth of our study trees by measuring tree height when plants were 4 years old and 8 years old and dividing by age to derive a growth rate.

### 2.5 Statistical analyses and calculations

To test if tree species vary in support of biodiversity, we first summed the abundance of each beetle species for each tree across the four sampling dates and then calculated beetle abundance,

richness, and Shannon index of diversity for each tree. Shannon index of diversity (H) was calculated using the formula:

$$H^{\prime }=‐\sum pi\;\ln pi$$

where *p_i_* is the proportion of individuals found in species *i*. We then used a generalized linear model (GLM) with tree species as predictor to test for significant differences among tree species in abundance, richness, and Shannon index of diversity of beetles. As both abundance and richness are expressed in counts, we fitted the model with Poisson distribution. Shannon index of diversity conformed to assumptions of normality and was hence fitted with normal distribution.

To test whether variation in support of biodiversity was based by a general beetle preference for plant traits, we first conducted principal component analyses to explore relationships among plant traits (N, P, K, SLA, and WD) and growth rate, and established a loading matrix for the two principal components describing most of the variation among tree species. Then, we tested if there was a relationship between either one of the two principal components and our beetle biodiversity metrics (abundance, richness, and Shannon richness) using correlation analyses. In cases where the relationship between a principle component and biodiversity was significant, we then used the three traits with the highest loading on that component in further correlation analyses. As other studies have shown that trait to function correlations may vary between different functional groups of plants (Gomes et al., 2021), we separated tree species in two functionally different groups, dipterocarps and non‐dipterocarps, and performed correlation analyses on each group separately. All of the above analyses were conducted in JMPpro 14.0.0 (SAS, 2018).

To estimate the level of plant diversity needed to capture most of the beetle richness and assess if 24 species were enough to capture most of the beetle richness, we used sample‐based rarefaction to establish smoothed species accumulation curves (Gotelli & Colwell, 2001) with 95% unconditional confidence intervals. This sample‐based approach for rarefaction is suitable in this context as we used tree species precisely analogous to samples. Through this we can estimate how adding species to a tree community contributes to overall beetle richness. This is also analogous to the concept of complementarity as defined as the gain in richness of adding some site to an existing set of sites (Williams et al., 2006) where sites in our case are represented by tree species. This is also an intuitive approach that is appropriate here as it is the number of tree species that restoration managers can control. Smoothed species accumulation curves were obtained by randomizing the order of tree species (i.e., samples) 1000 times (Longino & Colwell, 1997). In cases where our produced richness estimates reached a horizontal asymptote, this indicates that adding a new tree species will have little influence on richness. Nevertheless, for biodiversity studies in tropical habitats, such asymptotes may never be reached (Anderson & Ashe, 2000; Stork, 1991), but in such cases the curves themselves may be compared (Gotelli & Colwell, 2001). To assess how plant species with a disproportional support of associated biodiversity may influence richness of beetle communities, we also conducted the above estimations using the six tree species supporting the most species‐rich beetle communities. This cut‐off is based on the level of tree diversity that is typically manageable by restoration projects in Southeast Asia (sensu Benin et al. in review) where a large majority of restoration projects in the region use less than six species in operational restoration (mean of three species) (see also Chechina & Hamann, 2015). We also conducted sample‐based rarefaction to estimate how beetle species richness develops with extrapolated plant diversity. Based on Chao et al. (2014), extrapolation was limited to twice the level of plant diversity in the original sample. So for the rarefaction of the full tree community extrapolation ended at 48 tree species and in the six tree species community rarefaction extrapolation stopped at 12 tree species. The rarefaction procedures were done using EstimateS 9 (Colwell, 2005) that follows the analytical formulas of Colwell et al. (2004) and Colwell et al. (2012).

## 3 RESULTS

In sum, we collected 1417 individual beetles and found that the 24 tree species together supported 405 different species from 43 different beetle families. The collected community is also represented by a wide variety of different types of beetles; leaf‐chewing herbivores were the most common functional group among collected species and comprised 75% of all individuals collected (1061) followed by saprophagous (7%, 93), predators (6%, 90), taxa with mixed feeding (6%, 87), xylophagous, (4%, 55), and fungivores (2%, 31). Most of the beetles we collected were from the Chrysomelidae family with 38% (540) of all individuals collected and 31% (127) of all the species encountered (Table 1).

### 3.1 Tree species variation in support of beetle diversity

**FIGURE 2:**
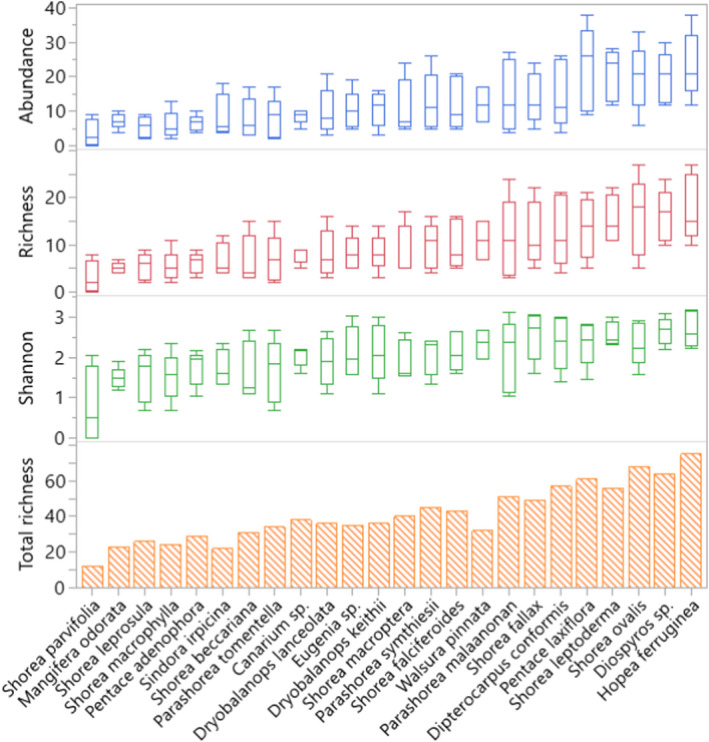
Box plot showing mean abundance, richness, and diversity (Shannon index) and bar plot of total richness of beetles (Coleoptera) sampled in the canopies of 8‐year‐old trees of 24 tropical tree species planted in a common garden in Sabah, Malaysia, Borneo. Generalized linear models indicate that beetle communities differed among tree species in abundance, species richness, and diversity (*p* < .0001, in all cases). Tree species are ordered in increasing mean richness. Species to the right can be considered particularly important for supporting beetle diversity

Agreeing with our first hypothesis, we found large and statistically significant variation among tree species in support of beetles in the mean abundance, mean richness, and Shannon index of diversity. Abundance varied 6.7‐fold and richness 5.9‐fold among different tree species (Figure 2) and GLM analyses show that these differences were statistically significant (*df* = 23, χ^2^ = 285.11, *p* < .0001, and *df* = 23, χ^2^ = 181.60, *p* < .0001, respectively). Shannon index of diversity also varied among tree species (*df* = 23, χ^2^ = 61.57, *p* < .0001). Tree species also varied 6.0‐fold in the total richness of beetles that they supported and total richness roughly resembled patterns of mean richness (Figure 2). Abundance, richness, and Shannon index of diversity were positively correlated in both dipterocarps (*p* < .0001) and in non‐dipterocarps (*p* < .002). We also found that the six species supporting the highest richness; *Hopea ferruginea*, *Diospyros* sp., *Shorea ovalis*, *Shorea leptoderma*, *Pentace laxiflora*, and *Dipterocarpus conformis*, together supported 62% of the total beetle biodiversity (253 of 405 species).

### 3.2 Plant economic spectrum as predictor of beetle diversity

**FIGURE 3:**
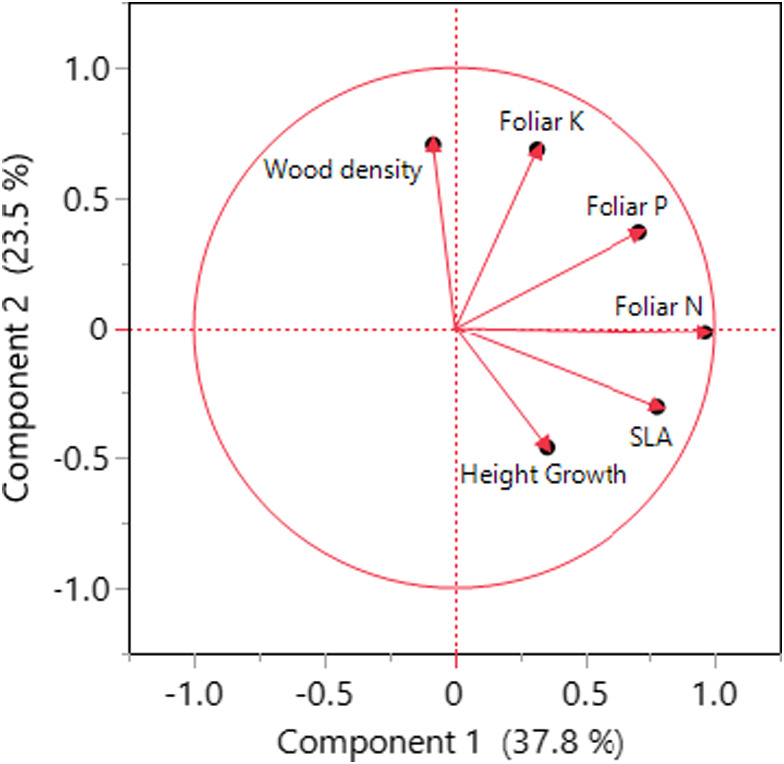
Simplifying relationships among six plant traits using principal component analysis indicates that 61% of the variation among 24 different tree species growing in a common garden in Sabah, Borneo, Malaysia, can be explained by two principal components. These characteristics are known to distribute plant species on the plant economic spectrum where plants with high nutritional status and SLA and light wood tend to result in high resource acquisition and growth (Baraloto et al., 2010)

Using a set of six plant characteristics (five plant traits and realized growth) that together are known to distribute plants along the acquisitive–conservative continuum, we found that 61.0% of the variation in traits among species could be described by two principal components (Figure 3). Principal component 1 (PC1) explained 37.8% of this variation and was primarily associated with leaf traits (i.e., foliar N and P, and SLA) that loaded high on PC1 (Table 2). Principal component 2 (PC2) explained 23.5% of the variation and was associated with wood density and growth rate, and also with foliar K (Table 2). Using the two principal components in correlation analyses, we found that it was primarily PC2 that was associated with beetle diversity (Table 3), that is, PC2 was significantly correlated with Shannon index of diversity (*r* = .5328, *p* = .0074) and to some extent also beetle richness (*r* = .3611, *p* = .0830). The traits explaining the association between PC2 and beetle diversity were not consistent across the full set of 24 tree species but differed between dipterocarps and non‐dipterocarps. In the dipterocarps, we found that a high Shannon index of diversity was correlated with slow growth (*r* = −.6119, *p* = .0090) and high wood density (*r* = .5151, *p* = .0343). High wood density was to some extent also associated with beetle richness (*r* = .4494, *p* = .0703) and beetle abundance (*r* = .4461, *p* = .0727). In the non‐dipterocarps, we found that foliar K was positively correlated with beetle diversity as expressed as Shannon index of diversity (*r* = .7511, *p* = .0516) and richness (*r* = .7782, *p* = .0393) but not beetle abundance (*r* = .5839, *p* = .1687). Beetle diversity was also close to significantly (*p* < .1) and positively correlated with growth rate in the non‐dipterocarps; richness (*r* = .7335, *p* = .0606), abundance (*r* = .6737, *p* = .0970), and Shannon index of diversity (*r* = .7074, *p* = .0754).

**TABLE 2:** Matrix showing the loading of six plant traits on two principal components explaining 61% of the variation among 24 different tree species growing in a common garden in Borneo, Sabah, Malaysia

	PC1	PC2
Foliar K	0.3126	**0.6865**
Foliar P	**0.7091**	0.3706
Foliar N	**0.9634**	−0.0172
SLA	**0.7797**	−0.3064
Wood density	−0.0849	**0.7052**
Growth rate	0.3513	**−0.4613**

Note

Bold values refer to the three traits with highest loading on each of the two principal components.

**TABLE 3:** Correlation between two principal components and beetle abundance, richness, and Shannon diversity index among 24 different tree species growing in a common garden in Borneo, Sabah, Malaysia

	PC1	PC2	Abundance	Richness	Diversity
PC1	1	<.0000	.2190	.2146	.0729
PC2		1	.2859	.**3611^†^**	.5328**
Abundance			11	.9681***	.8610***
Richness				1	.9155***
Diversity					1

Note

Values refers to correlation coefficients and bold letters indicate significant correlations (^†^ *p* < .10, *p < .05, **p < .01, ***p < .001).

### 3.3 Accumulation of beetle richness with increasing tree diversity

**FIGURE 4:**
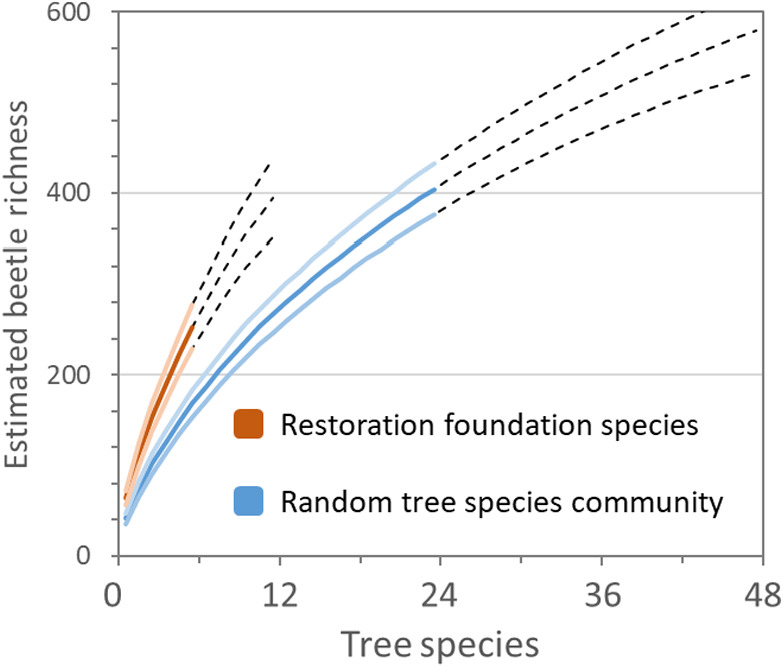
Smoothed accumulation curves of estimated beetle species richness (± 95% confidence interval) as a function of the number of tree species included in the tree species community, depending on if the community is based on a random collection of tree species (blue) or a selected set of six tree species with a disproportional support of high beetle richness (red). Colored solid lines represent the beetle richness based on observations, and black dashed lines are estimates from extrapolation. Following recommendations, extrapolation was constrained to double the sample size of the original tree species community

We used species accumulation curves to assess the functional relationship between plant species richness and beetle richness to estimate the level of plant diversity needed to rebuild canopy insect diversity in degraded secondary tropical forests. We found that a random community of 24 tree species was far from enough to reach asymptotic levels of beetle richness (Figure 4) and adding one additional tree species to a community of 24 tree species will, on average, increase beetle richness by 9.5 species. This estimate is analogous to the concept of complementarity (Williams et al., 2006) by estimating in what way adding yet another tree species to a community influences beetle richness. Extrapolation based on these 24 species also suggests that beetle richness continues to increase far beyond an extrapolation of twice the original number of tree species and that adding one additional tree species to a community of 47 will on average increase beetle richness by six species (Figure 4). Given that we were unable to reach an asymptote even from extrapolation, it is impossible to assess the plant diversity needed to cover most beetle diversity in this system.

We found that the six tree species supporting the most rich communities of beetles had a greater potential to support beetle diversity at lower plant species richness compared to a plant community of random species. This is shown by accumulation curves of the six selected species having a much steeper trajectory than the random tree species community (Figure 4). We found that community of six preferred tree species supported 33% higher richness of beetles compared to a community of six random tree species (223 species of beetles vs. 168).

## 4 DISCUSSION

The aim of this study was to establish a framework for evaluating if non‐random selection of tree species could be used as a starting point for restoring communities associated with trees in tropical forest restoration. If tree species vary in their importance for supporting biodiversity of associated communities and this variation can be predicted from their traits, then screening diverse forests can be accomplished without the need to intensively study biodiversity which is a more expensive and time‐consuming process than measuring traits. We found compelling evidence that tree species in tropical forests vary in their support of canopy beetles suggesting that by selecting tree species that harbor high diversity we can increase diversity and abundance over selecting random tree species. Because beetles make up large fraction of the arthropod biodiversity in tropical forests and include large functional variation, they are potential drivers of insect diversity and also higher trophic levels communities such as insectivorous birds and mammals (Hails & Kavanagh, 2013). Although other taxa and functional groups of tree‐associated organisms could be evaluated for conformance to tree species patterns of diversity, because beetles are the most diverse order of insects, we suggest that they represent a much needed starting point for rebuilding biodiversity in tropical forest restoration. The recent evidence of global insect collapse suggests that biodiversity triage during reforestation should proceed as a top priority and that models such as the trait‐based model presented here have high potential to guide the way.

We found that traits commonly used to describe plant species along the acquisitive–conservative trait continuum may be useful for predicting the diversity of associated communities. Hence, our research highlights that a trait‐based approach for screening tree species with the potential to support a high diversity of associated organisms for use in restoration is an effective framework. The idiosyncrasy of plant functional group variation in which certain traits support higher diversity than others is potentially explained by plant functional group variation in trade‐offs between traits, growth and herbivore defense, and how this influences the interspecific interactions within and among tropic levels. For the non‐dipterocarp tree species, beetles showed fidelity to plants with the acquisitive traits of fast growth and high foliar nutrients, especially foliar K. While some research suggests that fast‐growing plants may attract more herbivores due to the high nutritional value of acquisitive leaves or reduced chemical defenses in fast‐growing plants (Mooney et al., 2010; Price, 1991), fast growth itself may be a trait valued by herbivores indicating ability to maintain cellular turgor and rapid transpiration, both related to water availability to herbivores (Grady et al., 2013). In this perspective, it is also interesting to note that potassium is known as a fundamental plant compound that is indicative of plant health and influence a range of functions including tolerance to abiotic stress (Cakmak, 2005; Egilla et al., 2001; Santos et al., 2021). For the dipterocarp tree species, beetles showed a preference for host plants with the conservative resource allocation strategies of slow growth and high wood density, which is interesting as it did not line up with our expectations. Although we have no data to assess variation in defensive chemistry, we expect slow‐growing tree species to be well defended (Mooney et al., 2010; Price, 1991) which could make such trees less attractive to particular herbivores. Even if this might have been the case for some beetles, this was evidently not the case for our beetle community in which one‐fourth of the individuals were non‐herbivores. Interspecific interactions within and across tropic level may interact with plant nutrition and defense to influence the biodiversity associated with a tree (Fagundes et al., 2020). The influence of biotic interactions for biodiversity patterns is also expected to be particular influential in tropical forests (Schemske et al., 2009). Furthermore, an alternative hypothesis of the influence of plant defense on biodiversity is that plant defense may result in evolutionary counter adaptation and specialization in herbivores, and hence influence the level of diversity associated with a particular plant. A greater specialization of herbivores toward plant defense has also been suggested as one possible explanation to why tropical forests are so species rich (Peguero et al., 2017). As we found that slow‐growing dipterocarps with high wood density attracted higher abundance and richness of beetles, it follows that such species may be particularly important for promoting biodiversity in dipterocarp‐dominated forests.

With the enormous diversity contained in tropical forests, it is challenging to identify tree species that have a disproportional capacity to support biodiversity to be used in the restoration of tropical forests. In this study, we sampled canopy invertebrates four times throughout one season to assess the broader contribution of each tree species for beetle diversity. We acknowledge that this is a labor‐intensive approach that may not be feasible in many restoration operations. However, we found that tree species with a particular high conservation value can be identified using a trait screening process (see also; Peters et al., 2016). This trait screening is a promising applied research direction and could benefit by testing if certain traits consistently predict associated biodiversity in multiple tropical forests and in different biomes. In this study, it seems like plant characteristics were a better predictor of beetle diversity than phylogeny at least among the dipterocarps, that is, we found that the nine different species from the genus Shorea represented in this study were associated with very different levels of beetle diversity. Nevertheless, as we found that the position along the acquisitive–conservative continuum seems to have opposite effects on beetle diversity in dipterocarps vs. non‐dipterocarps, it seems there might be a need to consider functional belonging in selecting tree species by traits. Furthermore, as we also found that richness and abundance were positively correlated, it is possible that the value of a particular tree species for conservation could be predicted from simple assessment of abundance. Further studies using more diverse groups of canopy organisms and using different species selection methods could provide a better understanding of how to promote biodiversity during reforestation. This understanding would also benefit from studies assessing the trait to biodiversity relationship as trees mature and grow into canopy trees.

Previous studies have made great advancements in our understanding of broader patterns of canopy diversity in tropical forests by highlighting how a general lack of host specificity influences species turnover across hosts (Basset, 1999; Novotny et al., 2002; Wardhaugh et al., 2013). For example, findings of low host specificity of insect herbivores in tropical forests have reduced global estimates of arthropod diversity from 31 million to 4–6 million species (Novotny et al., 2002). Our study adds to this understanding by showing that tropical tree species vary in the abundance and richness of beetles they harbor suggesting that biodiversity may have a preference for certain plants over others. Such groupings of preferred hosts would not comprise species‐specific interactions, but may still confer some evolutionary significance to the group overall – such as has been shown in studies of diffuse co‐evolution (Thompson, 2005). Hence, understanding patterns of host preference are important for providing a better picture of how plant diversity relates to broader patterns of biodiversity in tropical forests.

Clearly, the conservation value of a particular tree species is related not only to richness but depends also on community composition and complementarity in the communities it support (Williams et al., 2006). We used rarefaction curves to assess this and as far as our extrapolation goes we found that complementarity was quite substantial. Adding yet another tree species to a community of 47 would on average add another six species of beetles to the community. This complementarity also mean that we never reached an asymptote level of diversity even from extrapolation. We are hence unable to estimate the level of tree diversity needed to support the larger majority of beetle species in this system. This is often the case in hyper‐diverse systems such as tropical forests (Anderson & Ashe, 2000; Stork, 1991). Nevertheless, even in cases when curves do not reach an asymptote, the curves themselves are still useful for assessing biodiversity patterns (Gotelli & Colwell, 2001).

We found that accumulation curves of beetle richness using six preferred tree species revealed steeper slopes compared to a random community of tree species implying that such species may indeed support richer communities at lower plant diversity. We found that six species of high conservation value would on average support 33% higher richness of beetles compared to random community of trees with the same number of species. Taken together, it follows that incorporating species that are disproportionally important for associated communities would be of great value for conservation – in particular, where low diversity plantings are used to maximize planting logistical efficiency, which is often the case (L. F. Benin, et al. (in review)) (Chechina & Hamann, 2015). Demonstrating that these differences are crucial as it relates to complementarity (Williams et al., 2006) and beta diversity. We could imagine a scenario in which a collection of tree species associated with a community that, despite having a high richness, may have similar composition. In such a case, we would not expect that adding new tree species to have a large influence on richness of the accumulated communities. Obviously, our study does not cover the full suite of biodiversity that may need consideration during restoration. Future studies should address if variation in support of associated communities is universal for a range of taxa, that is, are tree species that are preferred by beetle biodiversity also support biodiversity of other organisms. This is not well known but it is plausible that abundant communities of canopy insect may attract insectivorous birds and other wildlife (Hails & Kavanagh, 2013).

Although there are a number of examples where reforestation projects include a range of native tree species (Brancalion et al., 2018; Gustafsson et al., 2016; Schneider et al., 2014), most include only a handful of species of which the basic propagation characteristics are well known (Crouzeilles et al., 2017; Löf et al., 2019). A recent review report that a large majority of restoration projects in South East Asia use less than six species (mean of three) in operational planting (L. F. Benin et al. in review). Our findings suggest that such low‐diversity efforts are unlikely to cover the biodiversity of tropical forests. This is in line with findings that even with a strategy of high‐diversity plantings, restoration did not cover important functions provided by remnant forests in Brazil (Brancalion et al., 2018). By targeting tree species with a disproportional importance for associated biodiversity, it may be possible to maximize canopy biodiversity during reforestation. Given the current global commitment to use tree planting as a way to combat climate change, for example, via the trillion trees campaign, it seems that much more emphasis could be given to limiting the scale of the sixth mass extinction by explicitly trying to build biodiversity during reforestation. While we acknowledge that beetle diversity is not the only metric of canopy diversity, we suggest that the framework that we are building, a toolbox for rebuilding biodiversity using tree species with a disproportional importance for associated organisms, is an essential and much neglected aspect of global reforestation.

In our study, we relied on morpho‐species for assessment of biodiversity, which, in many cases, is the only feasible option in biodiversity assessments in tropical forests where many invertebrate species are unrecorded and not described (Barratt et al., 2003; Derraik et al., 2002). We acknowledge that this approach may underestimate species richness due to lumping (Derraik et al., 2002), that is, some beetle species may only be separated through detailed dissection or DNA analyses that may not be detected by morpho‐species determination. For the same reason, our approach may overestimate the average host use range, and underestimate the level when asymptote levels of richness are reached. Nevertheless, Barratt et al. (2003) found that richness estimates of beetles using morpho‐species were within about 10% of the actual number. Furthermore, as our sampling represents communities associated with the canopies of particular tree species, but do not directly assess host usage, it is plausible that some species would require additional plant diversity to sustain populations. This would be the case if beetle larvae require different plant species than adults that we assessed here, or if part of the sampled community is composed of transient species that actually need another host. This would result in overestimations of the level of diversity that can be sustained by a certain level of plant diversity.

## 5 CONCLUSIONS

Our results suggest that rebuilding diversity in tropical forests will require more plant species than are typically used in current reforestation and that if the goal is to rebuild biodiversity, such efforts could benefit by explicitly considering variation among tree species in conservation value. As we never reached an asymptote of beetle diversity, even from extrapolation two times the richness of the original plant community, we were unable to estimate how many plant species are needed in restoration to support maximal beetle diversity in our system. Clearly, the number needs to be higher than 48. Hence, identifying tree species with disproportional capacity to support biodiversity of the associated organisms and inclusion of such species in reforestation can enhance the contribution of enrichment planting to biodiversity, especially in low‐diversity plantings. Our study suggests that tree species selection toward this end can be directed trough a trait screening process. We see it as our goal as a society to ward off the sixth mass extinction, and more studies like ours that grapple with this issue are needed.

## CONFLICT OF INTEREST

The authors declare no competing interests.

## AUTHOR CONTRIBUTIONS

**Erik Petter Axelsson:**Conceptualization (equal); Data curation (supporting); Formal analysis (lead); Funding acquisition (equal); Investigation (equal); Methodology (equal); Supervision (equal); Validation (equal); Visualization (lead); Writing – original draft (lead); Writing – review & editing (equal). **Jane Vanessa Abin:** Data curation (lead); Investigation (equal); Validation (equal); Writing – review & editing (equal). **Maria Lourdes T Lardizabal:** Data curation (equal); Funding acquisition (equal); Investigation (equal); Project administration (equal); Resources (equal); Supervision (lead); Writing – review & editing (equal). **Ulrik Ilstedt:** Funding acquisition (equal); Project administration (equal); Resources (equal); Supervision (supporting); Writing – review & editing (equal). **Kevin C. Grady:** Conceptualization (equal); Funding acquisition (equal); Investigation (equal); Methodology (equal); Supervision (equal); Writing – original draft (supporting); Writing – review & editing (equal).

## Data Availability

The dataset used in this study is publicly available in Dyrad Digital Repository (https://doi.org/10.5061/dryad.gxd2547pc).
